# Efficacy and safety profile of elexacaftor-tezacaftor-ivacaftor triple therapy on cystic fibrosis: a systematic review and single arm meta-analysis

**DOI:** 10.3389/fphar.2023.1275470

**Published:** 2023-12-14

**Authors:** Wenye Xu, Ting Wu, Zijing Zhou, Zhihong Zuo

**Affiliations:** ^1^ Department of Critical Care Medicine, Xiangya Hospital, Central South University, Changsha, Hunan, China; ^2^ Hunan Key Laboratory of Molecular Precision Medicine, Department of Critical Care Medicine, Xiangya Hospital, Central South University, Changsha, Hunan, China; ^3^ Department of Cardiovascular Medicine, Xiangya Hospital, Central South University, Changsha, Hunan, China; ^4^ Department of Cardiovascular Medicine, The Third Xiangya Hospital, Central South University, Changsha, Hunan, China

**Keywords:** elexacaftor-tezacaftor-ivacaftor, cystic fibrosis, pulmonary function, sweat chloride, adverse events

## Abstract

**Background:** Elexacaftor-Tezacaftor-Ivacaftor (ELE/TEZ/IVA) is believed to be an effective and well-tolerated treatment for cystic fibrosis (CF), but the exact efficacy and safety profile are still unknown.

**Objective:** This study aimed to clarify the extent of functional restoration when patients are given with triple combination treatment and demonstrate the prevalence of adverse events, to evaluate the overall profile of ELE/TEZ/IVA on CF.

**Methods:** A literature search was conducted in PubMed, Web of Science and Cochrane Library. Random effects single-arm meta-analysis was performed to decipher the basal characteristics of CF, the improvement and safety profile after ELE/TEZ/IVA treatment.

**Results:** A total 53 studies were included in this analysis. For all the patients in included studies. 4 weeks after ELE/TEZ/IVA treatment, the increasement of percentage of predicted Forced Expiratory Volume in the first second (ppFEV_1_) was 9.23% (95%CI, 7.77%–10.70%), the change of percentage of predicted Forced Vital Capacity (ppFVC) was 7.67% (95%CI, 2.15%–13.20%), and the absolute change of Cystic Fibrosis Questionnaire–Revised (CFQ-R) score was 21.46 points (95%CI, 18.26–24.67 points). The Sweat chloride (SwCl) was significantly decreased with the absolute change of −41.82 mmol/L (95%CI, −44.38 to −39.25 mmol/L). 24 weeks after treatment, the increasement of ppFEV_1_ was 12.57% (95%CI, 11.24%–13.90%), the increasement of ppFVC was 10.44% (95%CI, 7.26%–13.63%), and the absolute change of CFQ-R score was 19.29 points (95%CI, 17.19–21.39 points). The SwCl was significantly decreased with the absolute change of −51.53 mmol/L (95%CI, −56.12 to −46.94 mmol/L). The lung clearance index_2.5_ (LCI_2.5_) was also decreased by 1.74 units (95%CI, −2.42 to −1.07 units). The body mass index increased by 1.23 kg/m^2^ (95%CI, 0.89–1.57 kg/m^2^). As for adverse events, 0.824 (95%CI, 0.769–0.879) occurred during ELE/TEZ/IVA period, while the incidence of severe adverse events was 0.066 (95%CI, 0.028–0.104).

**Conclusion:** ELE/TEZ/IVA is a highly effective strategy and relatively safe for CF patients and needs to be sustained to achieve better efficacy.

**Systematic Review Registration:** Identifier: CRD42023441840.

## Introduction

Cystic fibrosis (CF), an autosomal recessive disease most commonly in Caucasian populations, limits and shortens an individual’s life, in which the main cause of mortality is respiratory failure secondary to end-stage lung disease. It has an impact on multi-organs, with clinical manifestations including liver dysfunction, pancreatic insufficiency, malnutrition, increase of sweat chloride and the end-stage respiratory failure. Currently, early diagnosis through newborn screening, multi-divisional professional medical care, the strategy of medication, and availability of therapies are necessary for improving the survival rate of patients.

The pathogenesis of cystic fibrosis is now clearly proposed as the mutation of the Cystic Fibrosis Transmembrane Conductance Regulator (CFTR) gene, which was first described in 1989 (Berger et al., 1991). A low conductance cAMP-dependent chloride channel encoded by *CFTR*, is located at apical membrane of epithelial cells in several tissues, including respiratory tract, the gastro-intestinal tract, sweat glands duct, and the male reproductive tract (Crawford et al., 1991). *CFTR* mutations are divided into 6 groups, in which the p.Phe508del is class II and the most prevalent. p.Phe508del contributes to protein incorrectly folded and rapidly degraded, impairing protein trafficking to the cell surface and results in severe reduction of CFTR activity. In class I mutations, the production of CFTR protein is decreased; in class III mutations, the CFTR protein is not functional (“gating mutations”–for example G551D mutation); in class IV mutations, ions transport is diminished; class V mutations produce inadequate quantities of the CFTR protein and class VI mutations produce a less stable CFTR protein (Crawford et al., 1991; Ren et al., 2018). The phenotype of CF caused by class I-III mutations compared to class IV-VI mutations is much severer. Frequently, the mutations lead to more than one mechanism of protein failure, thus belong to more than one class.

Traditional therapies were the management of symptoms including airway clearance, antibiotics, and nutritional support. Until recent years, researchers pay attention to the recovery of CFTR function (Burgener and Moss, 2018; Bell et al., 2020). CFTR function might be partially rescued by small molecules known as modulators, which contains potentiators and correctors, that increase conductance of the CFTR channel and improve CFTR trafficking to the cell surface, respectively (Bardin et al., 2021; Shteinberg et al., 2021). Ivacaftor, one of the potentiators, was proven to be effective in a III clinical trial, leading to significant pulmonary and nutritional improvements, which also gave clues for further precise medicine in CF (Sermet-Gaudelus, 2013). Correctors, such as tezacaftor (TEZ), lumacaftor, and the next-generation elexacaftor (ELE), correct p.Phe508del folding and trafficking defect (Haggie et al., 2017; Donaldson et al., 2018; Southern et al., 2018). The combination of Ivacaftor (IVA) and Lumacaftor was firstly approved by the North American and European regulatory agencies, resulting in a modest improvement of lung function and other clinical outcomes (Wainwright et al., 2015). Hence, combined molecule therapy is getting more and more attentions.

Afterwards, it was found that treatment with TEZ/IVA was effective in terms of lung function and resulted in a significantly lower rate of pulmonary exacerbations than placebo (Southern et al., 2020). Additionally, compared to TEZ/IVA treatment, triple combination of elexacaftor-tezacaftor-ivacaftor (ELX/TEZ/IVA) has been shown to be the most effective strategy to improve the expression of corrected p.Phe508del CFTR protein at the cell surface according to *in vitro* experiments and clinical results (Furstova et al., 2022; Shakir et al., 2022; Pallenberg et al., 2023). Two *CFTR* correctors, ELX and TEZ, increase cell surface expression by improving folding and trafficking of F508del, and the *CFTR* potentiator IVA augments gating of CFTR channels inserted into the apical cell membrane (Wainwright et al., 2015; Taylor-Cousar et al., 2017). Triple combination showed unprecedented improvements in clinical outcomes including spirometry, nutritional outcomes, and patient-reported respiratory symptoms in patients with one or two F508del alleles (Zemanick et al., 2021). Significant reduction of sweat chloride concentration, a direct indicator of systemic CFTR function, was also demonstrated after triple therapy (Fidler et al., 2017). This triple combination was first approved in the United States of America in October 2019 for patients ≥12 years old, but has become available for children ≥6 years old since June 2021 (Goetz and Savant, 2021). However, the extent of functional restoration achieved by ELX/TEZ/IVA and the duration of this treatment strategy in these CF target organs in patients with F508del mutations has not been studied. The safety profile of this triple treatment remains to be elucidated as well.

In the systemic review and meta-analysis, we included 53 studies and analyzed the characteristics of patients, the endpoints, and adverse events after using ELE/TEZ/IVA, to evaluate the overall efficacy and safety.

## Materials and methods

This study was registered in PROSPERO, with registration No. CRD42023441840.

### Search strategy

We searched PubMed, Web of Science, and the Cochrane Library from 1998 to 4 August 2023. The following items were used: “elexacaftor-tezacaftor-ivacaftor”, “Trikafta” “VX445” “ELE/TEZ/IVA” and “cystic fibrosis” alone or in combination. The references of literature reviews and original articles were also scanned to avoid missing any qualified studies.

### Inclusion and exclusion criteria

The inclusion criteria were as follows: 1) prospective clinical studies (including randomized control trials and single-arm studies); 2) observational studies involving elexacaftor-tezacaftor-ivacaftor on cystic fibrosis; and 3) studies reporting the efficacy and safety of elexacaftor-tezacaftor-ivacaftor on cystic fibrosis. The exclusion criteria were as follows: 1) article type: letters, editorials, expert opinions, case reports and reviews; 2) studies without useable data; and 3) duplicate publications.

### Study Selection

All studies from the electronic search were uploaded into Endnote X9 and duplicates were removed. Two independent investigators reviewed remaining identified trials to confirm that they fulfilled the inclusion criteria. Finally, reference lists of included studies were screened to assess other potentially relevant studies. All disagreements were discussed and solved after rechecking the source data with a third investigator; in all cases one person recognized an error.

### Data extraction

Two investigators extracted data from the eligible studies independently, and any disagreements were resolved by discussion with a third investigator. For each study, the following characteristic information was recorded: the first author, year of publication, number of patients, CF related diabetes, pancreatic insufficiency, p.Phe508del mutation, percentage of predicted Forced Expiratory Volume in the first second (ppFEV_1_), percentage of predicted Forced Vital Capacity (ppFVC), lung clearance index_2.5_ (LCI_2.5_), Cystic Fibrosis Questionnaire–Revised (CFQ-R) respiratory domain score, sweat chloride concentration, body mass index (BMI), Hemoglobin A1C(HbA1c); absolute change in sweat chloride concentration from baseline at week 4 and week 24, absolute change in percentage of predicted FEV1 from baseline at week 4 and week 24, absolute change in percentage of predicted FVC from baseline through week 4 and week 24, absolute change in CFQ-R respiratory domain score from baseline at week 4 and week 24, absolute change in BMI from baseline at week 24, absolute change in LCI_2.5_ from baseline through week 24; the incidence of adverse events during elexacaftor-tezacaftor-ivacaftor treatment. Severe adverse events were defined by clinicians or investigators according to the severity of clinical manifestations in the included studies.

### Quality assessment

The quality assessment forms recommended by the Agency for Healthcare Research and Quality were used for quality assessment of the included trials. We resolved all disagreements through a discussion.

### Statistical analysis

The analysis software used in our study was Stata 14.0(Stata Corporation, College Station, Texas, United States of America). Meta-analysis was performed using the “metaprop” command. Since most of the included studies were single-arm studies, there may be high heterogeneity. Therefore, the combined results were expressed as the benefit value plus 95% confidence interval (CI) under the random effects model, and the incidence of each variable was analyzed. For all analysis results, *p* < 0.05 was considered statistically significant.

## Results

### Selection of included studies and study characteristics

**FIGURE 1:**
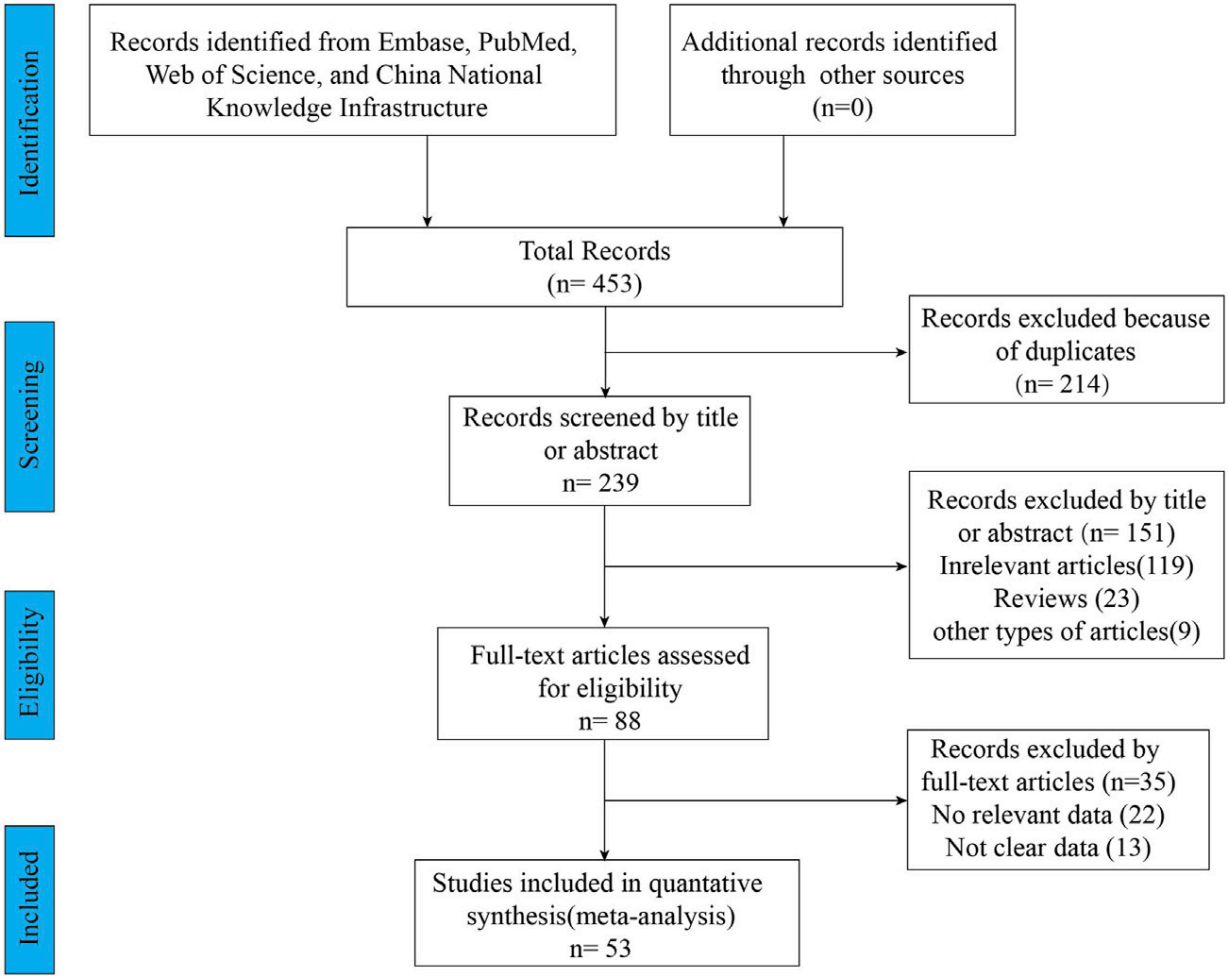
PRISMA diagram of study selection.

A total of 453 relevant articles were identified by searching online databases. Figure 1 showed the screening and selection process of the eligible trials. This meta-analysis included 53 studies, of which 11 articles were clinical trials and 42 articles were observational studies. All these studies were conducted in Caucasian countries. The age of included subjects ranged from 2 years old to 72 years old. The characteristics of the included trials were listed in Table 1. The results of the quality assessments were presented in Table 1.

**TABLE 1:** Study characteristics of included studies.

Author	Year	Country	Events (N)	Male (%)	Age min mean max	Research type	Quality
Heijerman et al. (2019)	2019	Belgium, Netherlands, United Kingdom, United States of America	55	44	12,/,/	RCT	High
Middleton et al. (2019b)	2019	North America, Europe, Australia	200	52	12,/,/	RCT	High
Shakir et al. (2022)	2022	United Kingdom	32	72	28, 32.5, 38.3	Prospective study	High
Burgel et al. (2021)	2021	France	245	55	24, 31, 38	Prospective study	High
Zemanick et al. (2021)	2020	North America, Europe, Australia	66	40.9	/, 9.3,/	RCT	High
DiMango et al. (2021)	2021	United Kingdom	43	33	/, 34,/	Prospective study	High
Djavid et al. (2021)	2021	United Kingdom	22	63.6	23, 37.2, 70	Retrospective study	Moderate
Griese et al. (2021)	2021	France	506	—	—	RCT	Moderate
Bermingham et al. (2021)	2021	United States of America	50	48	/, 32.0,/	Retrospective study	High
Crow et al. (2022)	2022	United States of America	12	27.3	/, 33.0,/	Retrospective study	Moderate
Berg et al. (2022)	2022	Denmark	50	44	18,/,/	Prospective study	High
Nichols et al. (2022)	2022	United States of America	487	49.5	/, 25.1,/	Prospective study	High
Carnovale et al. (2022b)	2022	Italy	20	40	/, 31.9,/	Retrospective study	Moderate
Spoletini et al. (2022)	2022	United Kingdom	13	23.1	/, 35,/	Retrospective study	Moderate
Goralski et al. (2022)	2022	United States of America	8	37.5	22, 32, 45	Prospective study	Moderate
Lee et al. (2023)	2022	Netherlands, United Kingdom United States of America	468	50.4	/, 26.41,/	RCT	High
Petersen et al. (2022)	2022	United States of America	134	54	/, 33.6,/	Retrospective study	High
Welsner et al. (2022)	2022	Ruhrlandklinik, Essen, Germany	29	52	20, 32, 49	Prospective study	High
Carnovale et al. (2021)	2022	Italy	47	42.5	17, 32, 52.6	Retrospective study	Moderate
Sutharsan et al. (2022)	2022	Australia, Belgium, Germany United Kingdom	87	51	/, 27.9,/	RCT	High
Mall et al. (2022)	2022	Australia, Canada, Denmark, France, Germany, Israel, Netherlands, Spain, Switzerland, and United Kingdom	60	41.7	/, 9.1,/	RCT	High
O'Shea et al. (2021)	2021	Irish	14	36	19, 34.4, 46	Retrospective study	Moderate
Salvatore et al. (2022)	2022	Italy	9	33.3	6.42, 9.75, 11.33	Retrospective study	High
Stapleton et al. (2022)	2022	United States of America	34	61.8	12, 27, 60	Prospective study	High
Carnovale et al. (2022a)	2022	Italy	26	53.8	20.8, 31.1, 48.3	Retrospective study	Moderate
Mainz et al. (2022)	2022	Germany, United Kingdom	107	49.5	/, 25.4,/	Prospective study	High
Zhang et al. (2022)	2022	United States of America	100	52	/, 35.3,/	Retrospective study	Moderate
Wucherpfennig et al. (2022)	2022	Germany	19	63.2	19, 31, 55	Retrospective study	Moderate
FitzMaurice et al. (2022)	2022	United Kingdom	24	46	/, 27,/	Prospective study	High
Stylemans et al. (2022)	2022	Belgium, United States of America	14	—	/, 36,/	Retrospective study	Moderate
Kos et al. (2022)	2022	Netherlands	20	37.8	12, 20.5,/	Prospective study	Moderate
Korten et al. (2022)	2022	Switzerland	16	37.5	13.0, 13.8, 15.4	Prospective study	Moderate
Martin et al. (2022)	2022	France	65	55.4	14, 32, 65	Prospective study	High
Scully et al. (2022)	2022	United States of America	23	44	/, 30.6,/	Prospective study	High
Walter and Bass (2022)	2022	United States of America	37	46	12, 30, 72	Retrospective study	Moderate
Barry et al. (2021)	2022	United Kingdom, Germany, Netherlands, United States of America	132	49.2	/, 37.7,/	RCT	High
Ramos et al. (2022)	2022	United States of America	94	45	31, 37, 45	Retrospective study	Moderate
McCoy et al. (2023)	2023	United States of America	18	56	15, 33.8, 49	Retrospective study	Moderate
Zhang et al. (2023)	2023	United States of America	56	58.9	/, 28.6,/	Prospective study	High
Appelt et al. (2023)	2023	Germany	25	24	/, 24.2,/	Retrospective study	Moderate
Piehler et al. (2023)	2023	Germany	70	48.6	/, 27.9,/	Prospective study	Moderate
Schnell et al. (2023a)	2023	Germany	69	61	12, 27.56, 56	Prospective study	Moderate
Cavinato et al. (2023)	2023	Italy	41	39.0	/, 29.91,/	Prospective study	Moderate
Lopes et al. (2023)	2023	Portugal	24	54.2	/, 26.9,/	Prospective study	High
Sheikh et al. (2023)	2023	United States of America	48	58.3	/, 28.8,/	Prospective study	Moderate
Steinack et al. (2023)	2023	Switzerland Germany	33	72.7	/, 27.8,/	Prospective study	High
Schnell et al. (2023b)	2023	Germany	20	75	/, 24.1,/	Prospective study	High
Tewkesbury et al. (2023)	2023	United Kingdom	267	65.1	/, 30.9,/	Retrospective study	Moderate
Nichols et al. (2023)	2023	United States of America	236	47.5	/, 24.8,/	Prospective study	High
Goralski et al. (2023)	2023	United States of America, Canada Australia, Germany	75	45.3	/, 4.1,/	RCT	High
Burgel et al. (2023)	2023	France	84	56.0	/, 30,/	RCT	High
Wainwright et al. (2023)	2023	United Kingdom, United States of America, Australia, Canada, Ireland	64	39.1	/, 9.3,/	RCT	High
Granados et al. (2023)	2023	United States of America	8	87.5	/, 22,/	Prospective study	Moderate

### The basic characteristics of pulmonary function, nutritional status, secretion of sweat glands in CF patients

**FIGURE 2:**
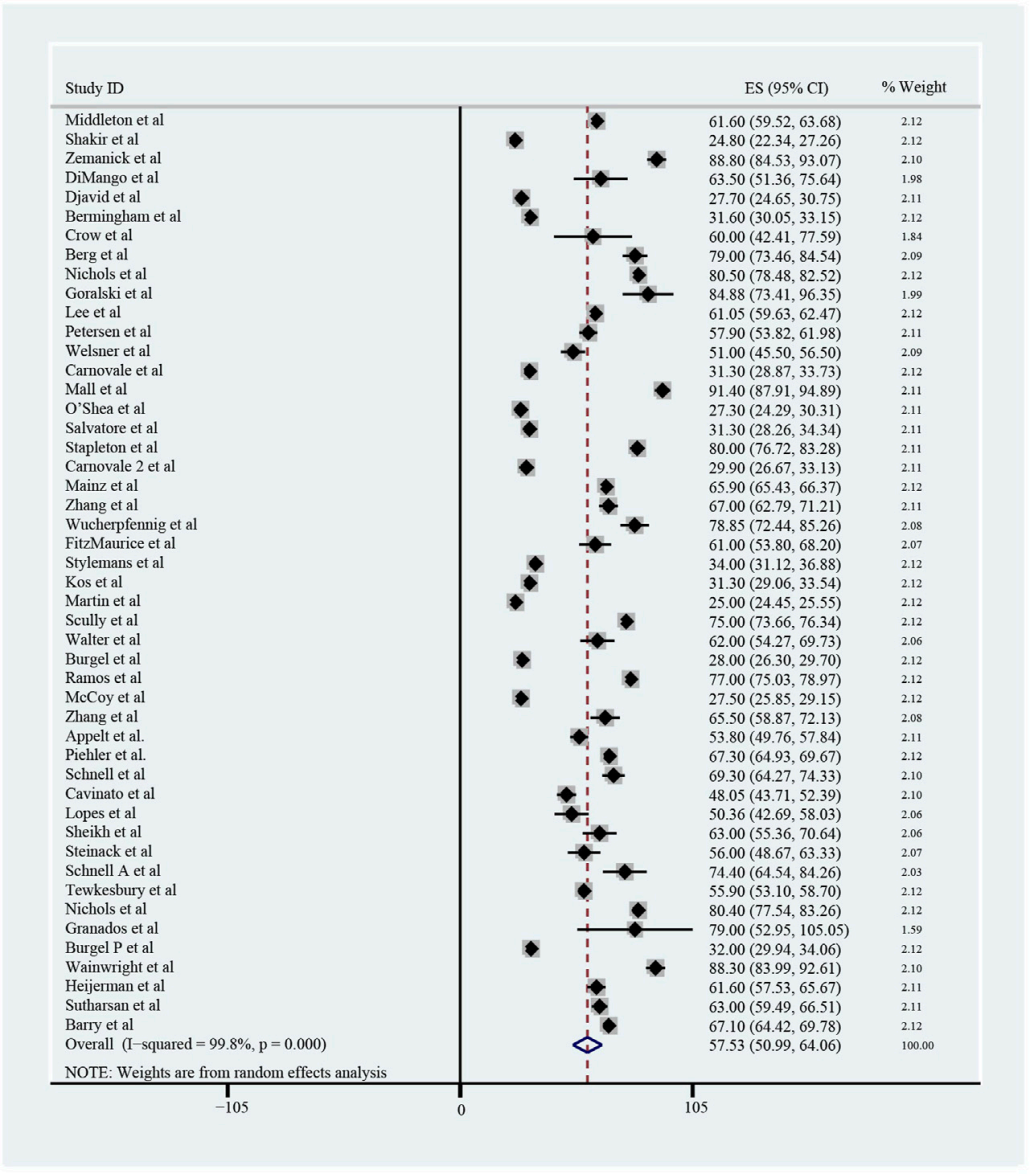
Percentage of Predicted Forced Expiratory Volume in the first second (ppFEV_1_) of included patients before ELX/TEZ/IVA treatment.

**FIGURE 3:**
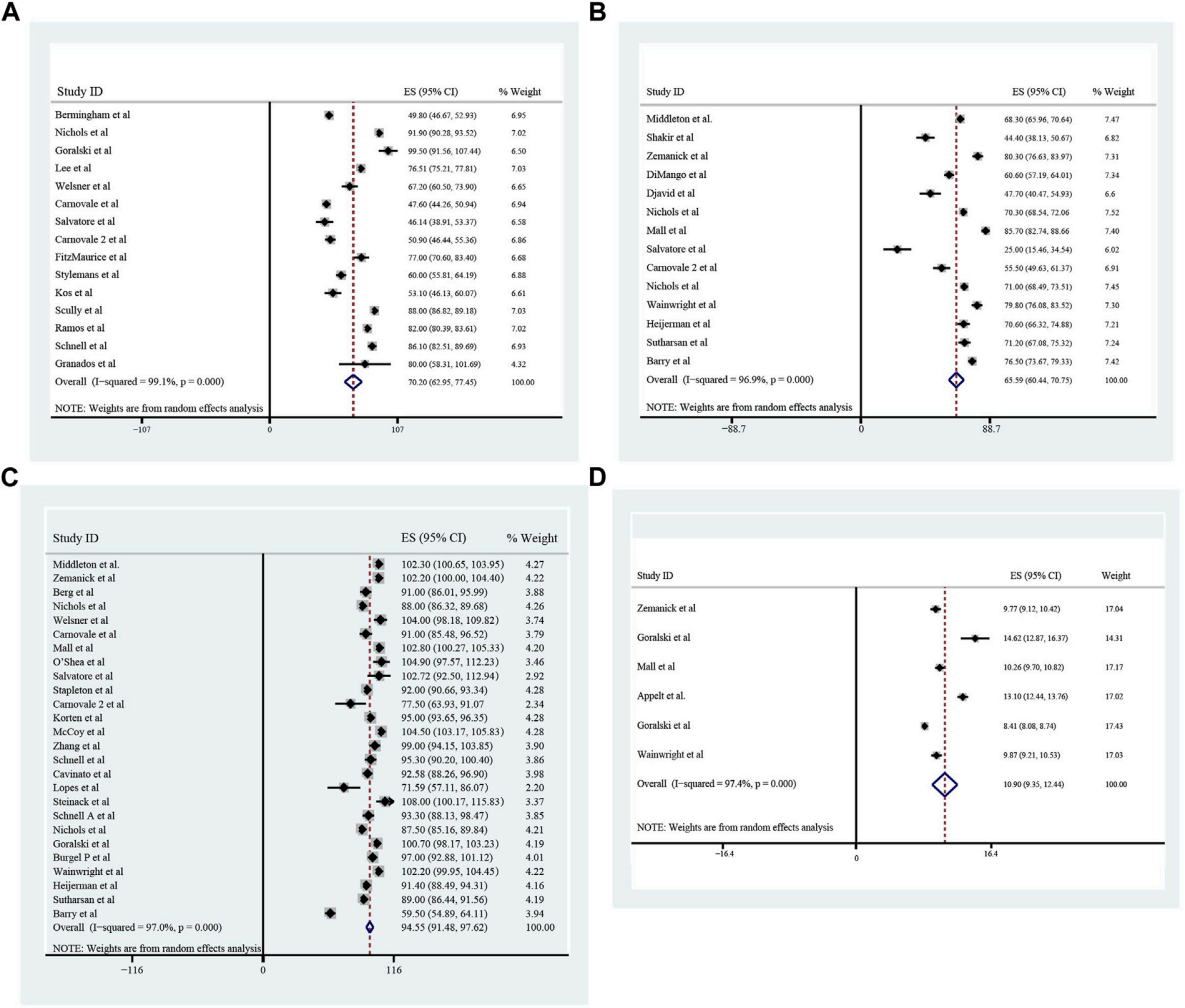
The Percentage of predicted Forced Vital Capacity **(A)**, Cystic Fibrosis Questionnaire-Revised Score **(B)**, Sweat Chloride **(C)**, and Lung Clearance Index_2.5_ **(D)** of included patients before ELX/TEZ/IVA treatment.

**FIGURE 4:**
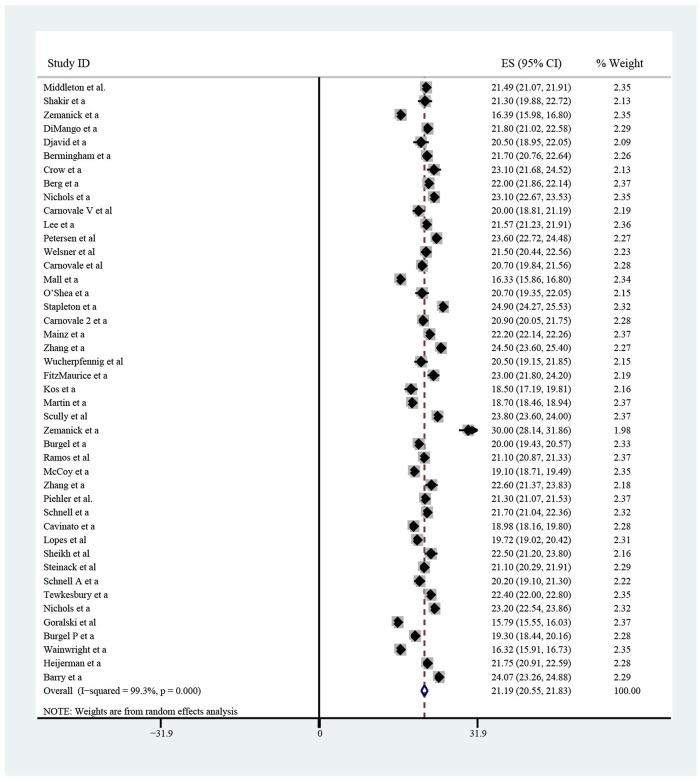
Body Mass Index of included patients before ELX/TEZ/IVA treatment.

**FIGURE 5:**
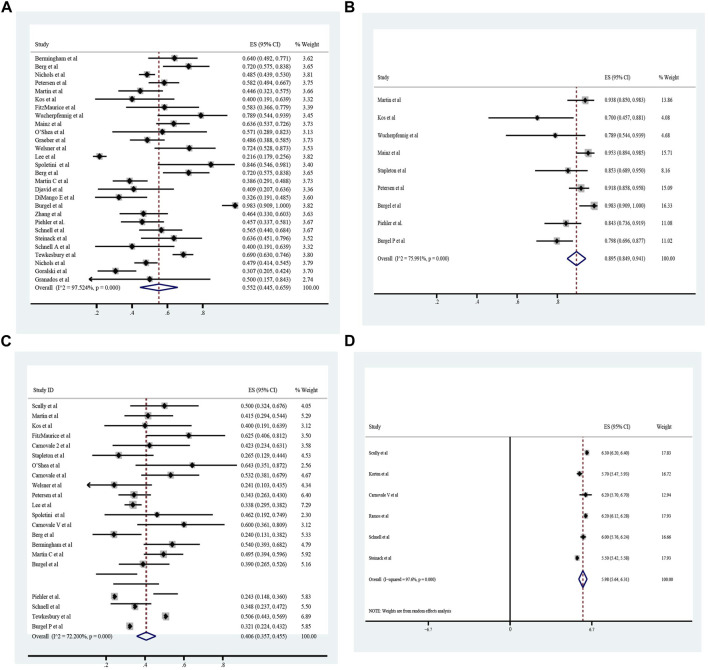
Homozygous F508 mutation **(A)**, pancreatic insufficiency **(B)**, cystic fibrosis related diabetes **(C)** and HbA1c **(D)** of included patients before ELX/TEZ/IVA treatment.

The pooled meta-analysis results indicated that ppFEV_1_ was 57.53% (95%CI, 50.99%–64.06%), ppFVC was 70.20% (95%CI, 62.95%–77.45%), CFQ-R score was 65.59 points (95%CI, 60.44–70.75 points), and LCI_2.5_ was 10.90 units (95%CI, 9.35–12.44 units), which revealed the overall pulmonary function of included patients. Patients with cystic fibrosis were inclined to suffer from diabetes, with the incidence of 0.406 (95%CI, 0.357–0.455). The HbA1c was 5.98% (95%CI, 5.64%–6.31%), close to the threshold limit value, which suggesting patients were at high risk of diabetes. Pancreatic insufficiency commonly occurred in 89.5% of CF patients, with 95%CI (0.849,0.941) in this meta-analysis. Among these patients, over a half were F508del homozygous, the incidence was 0.552 (95%CI, 0.445–0.659). As for the nutritional evaluation, the BMI of included subjects was 21.29 kg/m^2^ (95%CI, 20.55–21.83 kg/m^2^). SwCl, directly associated with the severity of CF, was 94.55 mmol/L (95%CI, 91.48–97.42 mmol/L). All these data show the basal characteristics of CF patients, which were seen in Figures 2–5 and Table2.

**TABLE 2:** The general characteristics of included subjects.

	Overall (95%CI)		Incidence (95%CI)
ppFEV1 (%)	57.53 (50.99, 64.06)	F508del homozygous	0.552 (0.445, 0.659)
ppFVC (%)	70.20 (62.95, 77.45)	Pancreatic insufficiency	0.895 (0.849, 0.941)
CFQ-R (points)	65.59 (60.44, 70.75)	CF related diabetes	0.406 (0.357, 0.455)
LCI2.5 (units)	10.90 (9.35, 12.44)		
SwCl (mmol/L)	94.55 (91.48, 97.42)		
HbA1C (%)	5.98 (5.64, 6.31)		
BMI (kg/m^2^)	21.29 (20.55, 21.83)		

Abbreviations: ppFEV1, percentage of predicted Forced Expiratory Volume in the first second; ppFVC, percentage of predicted Forced Vital Capacity; CFQ-R, Cystic Fibrosis Questionnaire–Revised; LCI_2.5_, lung clearance index_2.5_; SwCl, sweat chloride; HbA1C, Hemoglobin A1C; BMI, body mass index; CF, cystic fibrosis.

### Improvement of pulmonary function, nutrition, sweat chloride after triple treatment with ETX/TEZ/IVA for 4 weeks and 24 weeks

**FIGURE 6:**
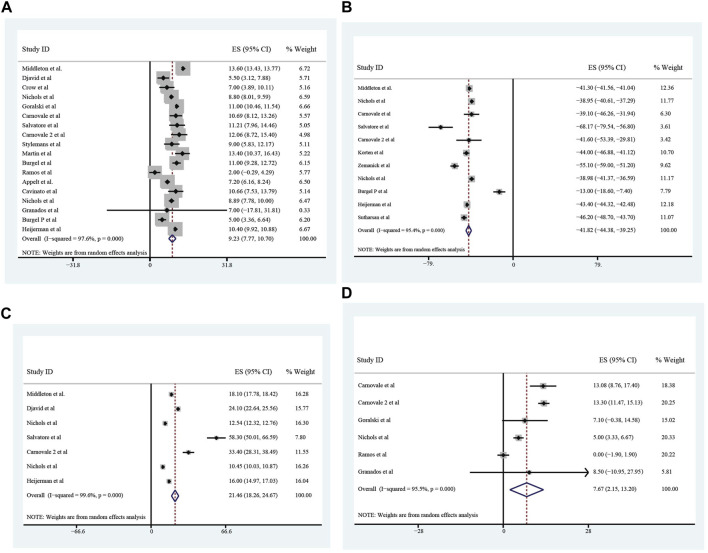
The absolute changes of Percentage of Predicted Forced Expiratory Volume in the first second **(A)**, Sweat Chloride **(B)**, Cystic Fibrosis Questionnaire-Revised Score **(C)**, and Percentage of predicted Forced Vital Capacity **(D)** among included patients after 4 weeks of ELX/TEZ/IVA treatment.

**FIGURE 7:**
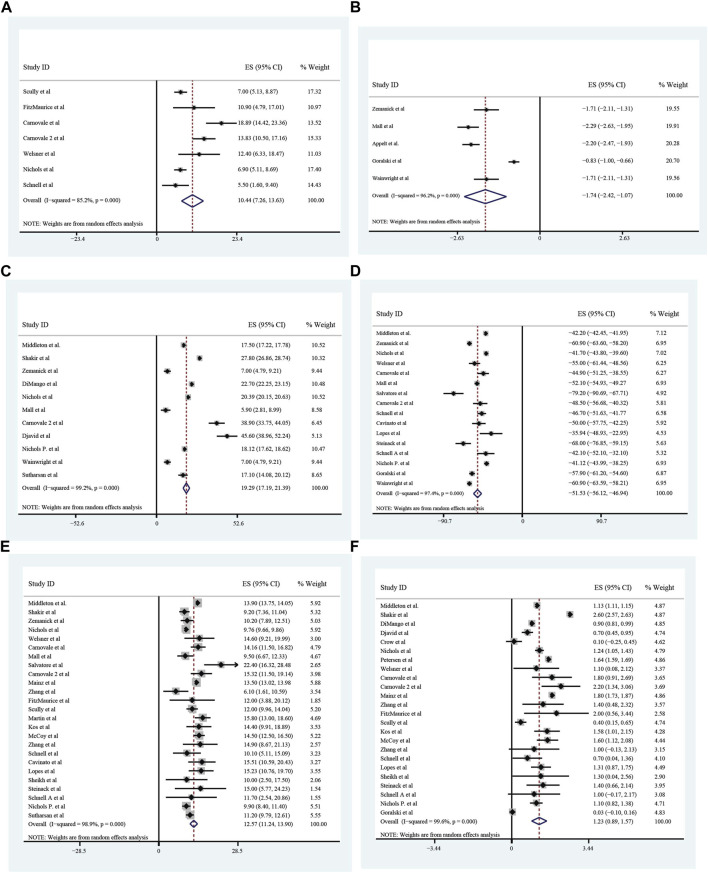
The absolute changes of Percentage of predicted Forced Vital Capacity **(A)**, Lung Clearance Index_2.5_ **(B)**, Cystic Fibrosis Questionnaire-Revised Score **(C)**, Sweat Chloride **(D)**, and Percentage of Predicted Forced Expiratory Volume in the first second **(E)** and Body Mass Index **(F)** among included patients after 24 weeks of ELX/TEZ/IVA treatment.

All these included patients were eligible for ETX/TEZ/IVA triple treatment. After being treated with triple combination for 4 weeks and 24 weeks respectively (Figures 6, 7), the pulmonary function, nutrition status and concentration of sweat chloride were improved at certain extent (Table 3). After 4 weeks, the increasement of ppFEV1 was 9.23% (95%CI, 7.77%–10.70%), the increasement of ppFVC was 7.67% (95%CI, 2.15%–13.20%), and the absolute change of CFQ-R was 21.46points (95%CI, 18.26–24.67points). The SwCl was significantly decreased with the absolute change at −41.82 mmol/L (95%CI, −44.38 to −39.25 mmol/L). At 24 weeks after treatment, the increasement of ppFEV1 was 12.57% (95%CI, 11.24%–13.90%), the increasement of ppFVC was 10.44% (95%CI, 7.26%–13.63%), and the absolute change of CFQ-R was 19.29points (95%CI, 17.19–21.39points). The SwCl was significantly decreased with the absolute change at −51.53 mmol/L (95%CI, −56.12 to −46.94 mmol/L). The LCI2.5 was also decreased with the value at −1.74units (95%CI, −2.42 to −1.07units). The BMI increased by 1.23 kg/m^2^ (95%CI, 0.89–1.57 kg/m^2^). All the data were shown in Table 3. Prolonged period of triple combination treatment contributes to better improvement.

**TABLE 3:** Improvement of lung function, nutrition and sweat chloride after triple treatment.

4 weeks after treatment		24 weeks after treatment	
	Overall (95%CI)		Overall (95%CI)
Absolute change of ppFEV1 (%)	9.23 (7.77, 10.70)	Absolute change of ppFEV1 (%)	12.57 (11.24, 13.90)
Absolute change of ppFVC (%)	7.67 (2.15, 13.20)	Absolute change of ppFVC (%)	10.44 (7.26, 13.63)
Absolute change of CFQ-R (points)	21.46 (18.26, 24.67)	Absolute change of CFQ-R (points)	19.29 (17.19, 21.39)
Absolute change of SwCl (mmol/L)	−41.82 (−44.38, −39.25)	Absolute change of SwCl (mmol/L)	−51.53 (−56.12, −46.94)
		Absolute change of LCI_2.5_ (units)	−1.74 (−2.42, −1.07)
		Absolute change of BMI (kg/m^2^)	1.23 (0.89, 1.57)

Abbreviations: ppFEV1, percentage of predicted Forced Expiratory Volume in the first second; ppFVC, percentage of predicted Forced Vital Capacity; CFQ-R, Cystic Fibrosis Questionnaire–Revised; SwCl, sweat chloride; LCI_2.5_, lung clearance index_2.5_; BMI, body mass index.

### The adverse events occurred during ETX/TEZ/IVA treatment

**FIGURE 8:**
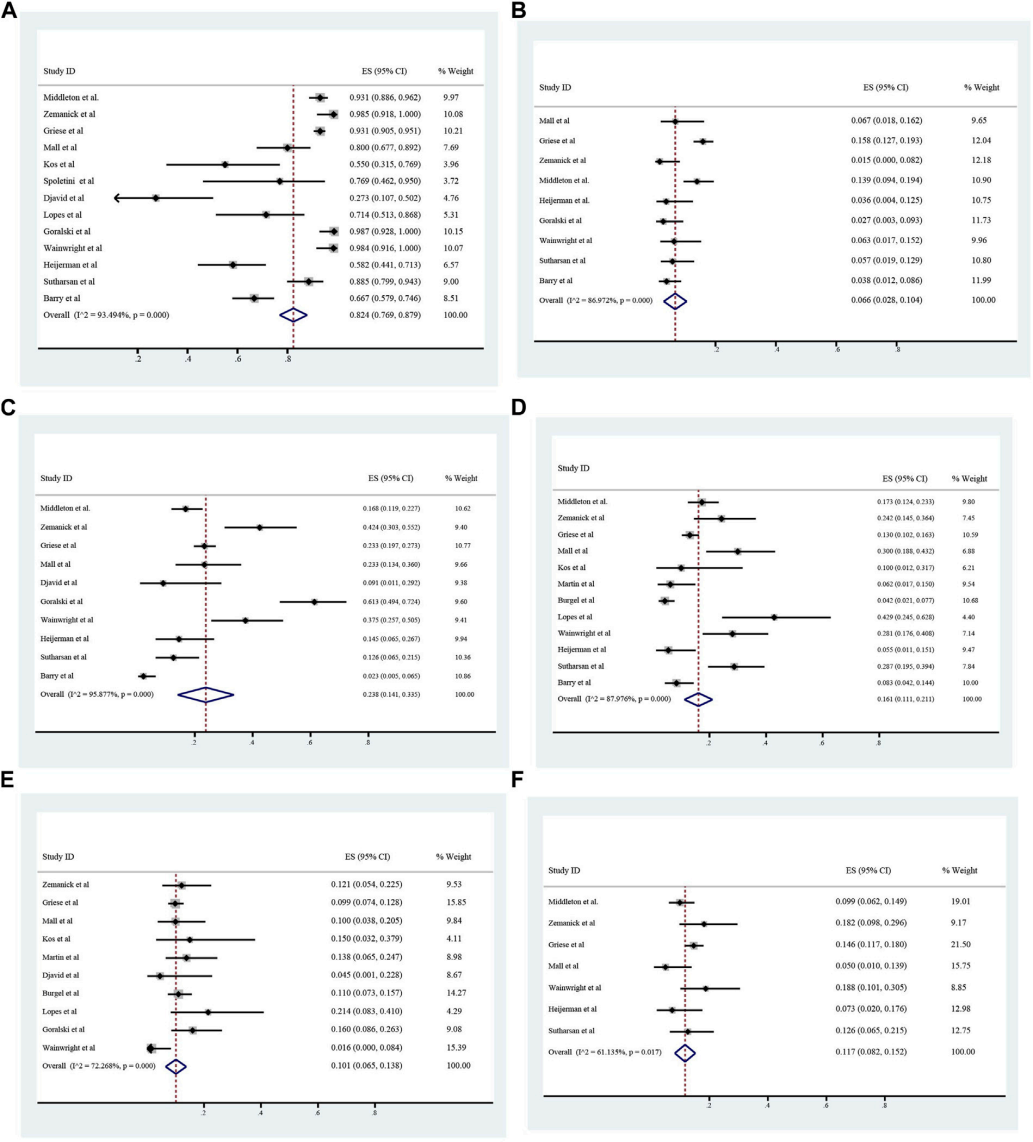
Incidence of total and severe adverse events **(A and B)**, cough **(C)**, headache **(D)**, rash **(E)**, and oropharyngeal pain **(F)** during ELX/TEZ/IVA therapy.

**FIGURE 9:**
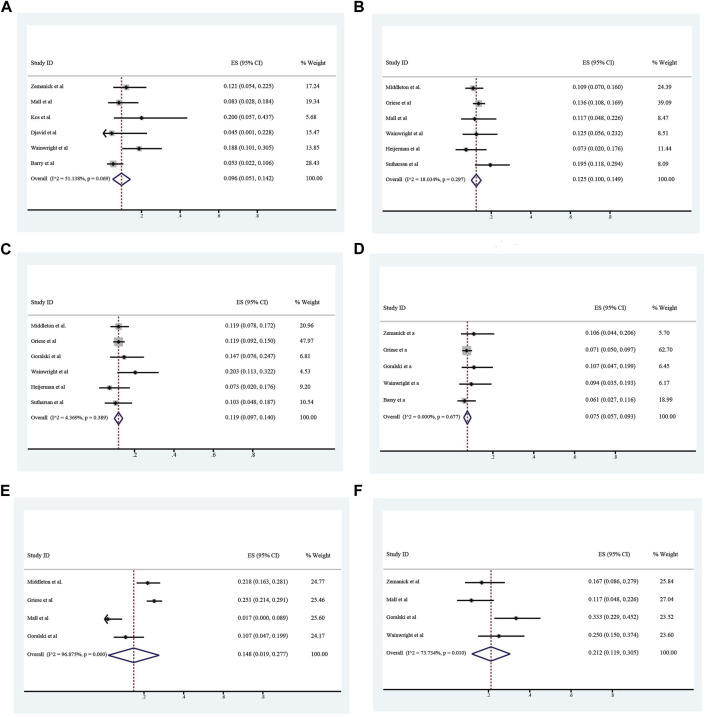
Incidence of abdominal pain **(A)**, nasopharyngeal pain **(B)**, upper respiratory tract infection **(C)**, alamine transferase increase **(D)**, infective pulmonary exacerbation of cystic fibrosis **(E)** and rhinorrhea **(F)** during ELX/TEZ/IVA therapy.

The overall incidence of adverse events associated with ETX/TEZ/IVA treatment was 0.824 (95%CI, 0.769–0.879), among which the incidence of severe adverse events was 0.066 (95%CI, 0.028–0.104). (Figures 8, 9).The specific symptoms of adverse events included cough 0.238 (95%CI, 0.141–0.335), rhinorrhea 0.212 (95%CI, 0.119–0.305), headache 0.161 (95%CI, 0.111–0.211), rash 0.101 (95%CI, 0.065–0.138), oropharyngeal pain 0.117 (95%CI, 0.082–0.152), abdominal pain 0.096 (95%CI, 0.051–0.142), nasopharyngitis 0.125 (95%CI, 0.100–0.149), upper respiratory tract infection 0.119 (95%CI, 0.097–0.140), transaminase increase 0.075 (95%CI, 0.057–0.093), infective pulmonary exacerbation of CF 0.148 (95%CI, 0.019–0.277).

## Discussion

In this single-arm meta-analysis, we included 53 studies in almost Caucasian countries, with the subjects ranging from children aged 2 years old to adults. For all the patients in included studies, 4 weeks after ELE/TEZ/IVA treatment, the increasement of percentage of ppFEV_1_ was 9.23% (95%CI, 7.77%–10.70%), the change of ppFVC was 7.67% (95%CI, 2.15%–13.20%), and the absolute change of CFQ-R score was 21.46 points (95%CI, 18.26–24.67 points). The SwCl was significantly decreased with the absolute change of −41.82 mmol/L (95%CI, −44.38 to −39.25 mmol/L). 24 weeks after treatment, the increasement of ppFEV_1_ was 12.57% (95%CI, 11.24%–13.90%), the increasement of ppFVC was 10.44% (95%CI, 7.26%–13.63%), and the absolute change of CFQ-R score was 19.29 points (95%CI, 17.19–21.39 points). The SwCl was significantly decreased with the absolute change of −51.53 mmol/L (95%CI, −56.12 to −46.94 mmol/L). The lung clearance index_2.5_ (LCI_2.5_) was also decreased by 1.74 units (95%CI, −2.42 to −1.07 units). The body mass index increased by 1.23 kg/m^2^ (95%CI, 0.89–1.57 kg/m^2^). Unsurprisingly, the prolonged course of treatment brings more improvement. Additionally, these triple combination led to an overall 82.4% incidence of adverse events, while the incidence of severe ones was only 6.6%. Thes adverse events were common symptoms, such as cough, headache, rash, and oropharyngeal pain, which were unlikely to cause medication discontinuation or disease exacerbation.

SwCl is believed to be directly associated with *CFTR* function. Different functional classes of CFTR mutations would have differences in epithelial chloride conductance, thus leading to variations of SwCl in children and adults (Wilschanski et al., 1995; Lebecque et al., 2002). Multicenter trials suggested SwCl as a biomarker of *CFTR* activity and to test the effect of CFTR potentiators (Accurso et al., 2014). Therefore, the test of SwCl is used as the gold standard for CF diagnosis among symptomatic patients, also within the infant screening and in the follow-up of CF patients during molecular therapies (Sontag, 2016). Although the sweat test is considered a robust measure, SwCl measurements in patients with CF and a G551D mutation had an inherent biological variability that was higher than commonly considered (Vermeulen et al., 2017). Notably, the normal SwCl could not exclude CF (Stewart et al., 1995). During infant screening, SwCl concentrations in CF patients did not change in a meaningful way during the first year of life (LeGrys et al., 2019). Additionally, compared to males, SwCl response was larger in females, which also explained the negative correlation of weight with the response in sweat chloride concentration after start of lumacaftor/ivacaftor (Aalbers et al., 2021). Furthermore, the value of SwCl also varied among different age (Traeger et al., 2014). Nevertheless, SwCl concentration did not necessarily predict a milder pulmonary course in patients with cystic fibrosis (Davis et al., 2004). *Heltshe* et al. found it was difficult to identify a minimum threshold for change in SwCl that was associated with FEV1 improvement (Heltshe et al., 2013). There was significant variability in sweat chloride distribution across CFTR class 2–5 genotypes. The relationship between sweat chloride and mortality varied by genotype with a relatively strong relationship in R117H/F508del patients (Espel et al., 2018). SwCl concentration might be a useful predictor of mortality and clinical phenotype when CFTR genotype functional class was unclassified (McKone et al., 2015). A clinical trial proposed SwCl was as a clinical endpoint to measure efficacy based on ivacaftor as well (Durmowicz et al., 2013). Changes in SwCl at early stage of treatment had the sufficient predictive potential to identify the individuals that showed large improvement at later stage (Seliger et al., 2013). ELX/TEZ/IVA treatment of children with CF led to greater improvements in sweat chloride than those previously seen in adults and adolescents (Middleton et al., 2019a; Shakir et al., 2022). Even though the concentration of SwCl is influenced by gender, age, and types of mutations and might be associated with lung function, both observational studies and clinical trials consider it as one of endpoints in evaluating therapy. As shown in this meta-analysis, regardless of age distribution, male percentage and previous medicine take, SwCl decreased by 41.82 mmol/L and 51.53 mmol/L after 4 weeks and 24 weeks respectively, which indicated triple treatment improved CFTR function efficiently and longer treatment duration brought about more reduction. Moreover, the decrease of SwCl was related to improved survival in patients with CF (McKone et al., 2015). In conclusion, substantial improvements in sweat chloride in response to ELX/TEZ/IVA therapy may presage improvements in long-term clinical outcomes.

Improvement of pulmonary function is another manifestation of molecular therapy. Lung is one of the major target organs of CF. In the worst scenario, patients would develop pulmonary fibrosis and require lung transplant or even die. Spirometric variables, FEV1 and FVC, are employed to measure the lung disease progression of CF. FEV1 has served as the critical primary endpoint of most clinical trials (Fuchs et al., 1994; Saiman et al., 2003). The rate of lung function decline was shown to be related to survival of CF (Corey et al., 1997; Schluchter et al., 2002), while decline in lung function did not predict future decline in CF patients (Rosenfeld et al., 2015). In a retrospective study, even with attenuated lung function decline, there were still reduced survival in adult patients (Keating et al., 2017). Hence, studies evaluated efficacy based on improvement of FEV1 and FVC rather than rate of decline. A decrease in ppFEV1 predicted a decrease in health-related quality of life over time, which demonstrated ppFEV1 should be increased to improve patients’ life quality (Abbott et al., 2013). The value of FEV1 at age 6 was proved to be an independent predictor for CF-related mortality by a retrospective study (Vandenbroucke et al., 2020). Also, a marked improvement of ppEFV1 occurred in survival patients with an FEV1 below 30% (George et al., 2011). Basically, children had a higher baseline of ppFEV1, ppFVC and CFQ-R score than adolescents and adults (Middleton et al., 2019a; Shakir et al., 2022). Despite different baselines, ELX/TEZ/IVA therapy led to a 9.23% improvement in mean ppFEV1, a 7.67% increase in mean ppFVC, and 21.67 point in CFQ-R score (a questionnaire based on patients’ self-evaluation) at week 4 in present study. LCI_2.5_, a measure of ventilation inhomogeneity that might be more sensitive than spirometry in detecting lung function changes during childhood, had a mean decrease of 1.74 units after 24-week triple therapy, which further corroborated the improvement of ppFEV1. LCI_2.5_ was proved to be able to discover early stage of lung injury in CF based on a large longitudinal study (Kraemer et al., 2005) and it was also indictive for the presence of lung structure changes after 3 years in 87% CF cases (Fuchs et al., 2014). Early initiation of ELX/TEZ/IVA in children with CF is helpful for improving lung function and minimizing the decline of ppFEV1 and LCI_2.5_ tightly associated with disease progression (VanDevanter et al., 2016). In addition, most studies focused on the efficacy on patients older than 6 years old, while a recent clinical trial found even in children 2 through 5 years of age, ELX/TEZ/IVA led to clinically meaningful reductions in SwCl concentration and lung clearance index (Goralski et al., 2023). Regardless of age, gender or mutation type, ELX/TEZ/IVA is an effective therapy, rapidly ameliorating the lung function in CF patients.

Benefits of ELX/TEZ/IVA combination were observed on other important endpoints, including surrogates for nutritional health. On one hand, CFRD, a complex and multifactorial disease, attributing to insulin secretion insufficiency and peripheral insulin resistance, represents capacity of dealing with carbohydrate. The mean prevalence of CRFD in this meta-analysis was 40.6% and HbA1c was 5.98%, close the upper range of reference. *CFTR* modulators were shown to be beneficial for controlling blood glucose level (Crow et al., 2022; Korten et al., 2022). However, relevant studies were not adequately conducted to quantify its efficacy. On the other hand, weight and BMI are accompanied with nutritional status; however, BMI is more commonly used to measure an individual’s nutrition. BMI in CF was closely correlated with improvements in lung function and was independent predictors of survival (Liou et al., 2001; Yen et al., 2013). Underweight status was independently associated with adverse clinical outcomes in CF, including worsening lung function, morbidity, and mortality (Culhane et al., 2013). Even for severe cases requiring lung transplantation, low BMI was suggested to portend a poor outcome after transplantation (Snell et al., 1998). CF management guidelines have focused primarily on attenuation of nutritional failure with recommendations to maintain a BMI above 22 kg/m^2^ in women and 23 kg/m^2^ in men (Castellani et al., 2018). Nevertheless, overweight and obesity were risk factors in adult CF patients with severe scenario (Harindhanavudhi et al., 2020), which was further validated in an Italy multicenter study (Gramegna et al., 2022). In addition, a meta-analysis also concluded that the currently recommended target BMI in patients with CF should be reconsidered, since higher BMI was not beneficial for outcomes of CF (Nagy et al., 2022). In present study, the mean BMI of included subjects is 21.29 kg/m^2^, and the increase of BMI after 24-week triple treatment is 1.23 kg/m^2^, indicating that ELX/TEZ/IVA is promising for improving patients’ nutrition status.

Adverse events are prevalent in ELX/TEZ/IVA triple therapy with an incidence at 0.824, which were almost associated with CF development and mild to moderate, while rate of severe adverse events was only 6.6%, rarely leading to the discontinuation of treatment. All clinical trials in present study concluded consistent results that ELX/TEZ/IVA was generally safe and well-tolerated. Cough, rhinorrhea, and headache ranked top 3 among these adverse events. The notable adverse events, infective pulmonary exacerbation of CF, occurred in 14.8% patients. Pulmonary exacerbations were associated with a higher risk of lung function decline and decreased survival (Waters et al., 2012). Compared to placebo treatment, the incidence of pulmonary exacerbations in children aged 6 to 11 receiving ELX/TEZ/IVA treatment was only 1.7% in a randomized clinical trial (Mall et al., 2022). However, in another clinical trial conducted in patients 12 years of age or older, pulmonary exacerbations in ELX/TEZ/IVA group was 21.8%, while in placebo group it was 47.3% (Middleton et al., 2019b). The differences between these clinical trials resulted from age and dosage of medicine. Even relative to the TEZ/IVA group, there was still a reduction in reported adverse events of infective pulmonary exacerbation of CF in the ELX/TEZ/IVA group (Shakir et al., 2022). Although commonly seen during the treatment, these adverse events were still under control and unlikely to cause deterioration of cystic fibrosis. Nevertheless, small size of samples in most included studies limited the ability to detect uncommon and rare adverse events, which were not categorized and shown in the analysis, for example, influenza. In summary, the overall safety profile of present study was consistent with clinical trials and real-world findings.

Among these studies included in this meta-analysis, most followed up the efficacy and safety after 4 and 24 weeks of ELX/TEZ/IVA treatment and patients achieved the initial improvement as early as 4 weeks after receiving medications. 48-week and 2-year period were investigated by several studies, and it was found no loss of pulmonary function or any new safety concern, suggesting ELX/TEZ/IVA is persistently effective and well-tolerated (Carnovale et al., 2022a; Lee et al., 2023). However, it is urgent to determine the exact follow-up periods to validate the observed improvements with respect to clinical stability, slowing down or stopping the decline in pulmonary function, reducing the incidence of complications, improving the survival and enhancing the compliance of patients. Further prospective research and clinical trials are needed to determine the optimal duration of therapy. Additionally, patients at different age would be prescribed for different dosage, bringing in the variance of improvement and safety events. Therefore, the best dosage for different age groups requires further exploration.

## Limitations

Due to ethical issues, the majority of clinical trials did not include a placebo group. The incidence of safety events attributable to the underlying disease process was not ascertained and the efficacy could not be compared quantitively and directly. Secondly, CF is prevalent in children to adult patients and has several mutations, so it is difficult to do sub-analysis for age and mutation, respectively. The mean incidence of homozygous F508del mutation in present meta-analysis is 55.2%, which predominated among all mutations. Moreover, most study follow-ups were conducted during the pandemic of COVID−19, and the safety results reported were based on both in-clinic and in-home safety assessments, while the in-home safety assessments were not presented in study.

## Conclusion

In conclusion, this single-arm meta-analysis demonstrated that ELX/TEZ/IVA therapy is effective and well-tolerated among CF patients in different age groups. Prolonged treatment duration would bring more improvement of pulmonary function, CFTR function and nutritional status without extra risk of adverse events. Clinicians are supposed to encourage the application of ELX/TEZ/IVA and adjust the dosage and period according to the individual’s variance.

## Data Availability

The original contributions presented in the study are included in the article/supplementary material, further inquiries can be directed to the corresponding author.
